# Selective Calcium Sensitivity in Immature Glioma Cancer Stem Cells

**DOI:** 10.1371/journal.pone.0115698

**Published:** 2014-12-22

**Authors:** Shimei Wee, Maria Niklasson, Voichita Dana Marinescu, Anna Segerman, Linnéa Schmidt, Annika Hermansson, Peter Dirks, Karin Forsberg-Nilsson, Bengt Westermark, Lene Uhrbom, Sten Linnarsson, Sven Nelander, Michael Andäng

**Affiliations:** 1 Department of Physiology and Pharmacology, Karolinska Institutet, 171 77 Stockholm, Sweden; 2 Department of Medical Biochemistry and Biophysics, Karolinska Institutet, 171 77 Stockholm, Sweden; 3 Department of Immunology, Genetics and Pathology, Uppsala University, 751 85 Uppsala, Sweden; 4 The Hospital for Sick Children, University of Toronto, Toronto, Canada; Johns Hopkins University, United States of America

## Abstract

Tumor-initiating cells are a subpopulation in aggressive cancers that exhibit traits shared with stem cells, including the ability to self-renew and differentiate, commonly referred to as stemness. In addition, such cells are resistant to chemo- and radiation therapy posing a therapeutic challenge. To uncover stemness-associated functions in glioma-initiating cells (GICs), transcriptome profiles were compared to neural stem cells (NSCs) and gene ontology analysis identified an enrichment of Ca^2+^ signaling genes in NSCs and the more stem-like (NSC-proximal) GICs. Functional analysis in a set of different GIC lines regarding sensitivity to disturbed homeostasis using A23187 and Thapsigargin, revealed that NSC-proximal GICs were more sensitive, corroborating the transcriptome data. Furthermore, Ca^2+^ drug sensitivity was reduced in GICs after differentiation, with most potent effect in the NSC-proximal GIC, supporting a stemness-associated Ca^2+^ sensitivity. NSCs and the NSC-proximal GIC line expressed a larger number of ion channels permeable to potassium, sodium and Ca^2+^. Conversely, a higher number of and higher expression levels of Ca^2+^ binding genes that may buffer Ca^2+^, were expressed in NSC-distal GICs. In particular, expression of the AMPA glutamate receptor subunit GRIA1, was found to associate with Ca^2+^ sensitive NSC-proximal GICs, and decreased as GICs differentiated along with reduced Ca^2+^ drug sensitivity. The correlation between high expression of Ca^2+^ channels (such as GRIA1) and sensitivity to Ca^2+^ drugs was confirmed in an additional nine novel GIC lines. Calcium drug sensitivity also correlated with expression of the NSC markers nestin (NES) and FABP7 (BLBP, brain lipid-binding protein) in this extended analysis. In summary, NSC-associated NES^+^/FABP7^+^/GRIA1^+^ GICs were selectively sensitive to disturbances in Ca^2+^ homeostasis, providing a potential target mechanism for eradication of an immature population of malignant cells.

## Introduction

Glioblastoma multiforme (GBM) is a highly malignant form of brain cancer with poor prognosis for affected individuals. Despite the combination of surgery, chemotherapy and radiotherapy, more than 90% of the patients show recurrence [1], and the median survival remains as low as 14–16 months [2]. Although malignant glioma tumors are highly heterogenous, a subpopulation of immature cells, termed glioma initiating cells (GICs) [2]–[6] coexist with more differentiated cell populations. GICs have been shown to be resistant to radio- and chemotherapy and are believed to be responsible for the tumor relapse [7]. Reflecting the immaturity of GICs and their ability to differentiate [8], these cells have been shown to share a stem cell (stemness)-associated gene expression with stem cell populations, such as teratoma-forming normal embryonic stem cells [9]–[11], and it is proposed that GICs continuously resupply the bulk tumor cells through self-renewal and differentiation [11], [12]. Much of the drug development research for GBM treatment has focused on targeting bulk cells, most of which lack tumor-initiating capacity. A major challenge that remains is increasing the efficacy of cancer treatment targeting GICs as these cells exhibit resistance to chemo- and radiotherapy using current strategies.

Although several signaling pathways such as Notch, Hedgehog-Gli, RTK-Akt, BMP/TGF-β, WNT-β-catenin and STAT3 have been shown to support self-renewal of stem cells and immature cancer cells [13], potential therapeutic targets that can selectively eradicate GICs are few [14]. An alternative strategy to render GICs less aggressive was demonstrated by BMP induced differentiation therapy [8]. Also dopamine D2 receptor antagonists have been identified to drive differentiation of relatively differentiation-resistant leukemic and breast tumor initiating cells [15].

Ion channels have long been assigned the role of governing basic cellular processes in addition to electrical excitability and for example potassium and Ca^2+^ channel signaling control diverse functions as proliferation and migration in stem cells and cancer cell lines [16], [17]. Ca^2+^ has also been implicated in cancer cell survival [18]. Recently, it was also shown that interference with a Ca^2+^ channel subunit was able to drive liver tumor-initiating cells into apoptosis [19].

In this study, we set to investigate mechanisms unique for the stemness-associated functions in glioma cells and conclude that stem-like cells are more sensitive to Ca^2+^ disturbances compared to more mature cell types.

## Materials and Methods

### Cell culture

GliNS1, G179NS and G166NS GIC lines were grown in culture as previously described [6], [11]. Briefly, the cells were first grown as spheres in the first week before transferring to laminin-coated dishes, where they were grown as adherent monolayers in serum-free human Neurocult NS-A basal media (StemCell Technologies) supplemented with Glutamax, Hepes, N2, B27 (Invitrogen), EGF (10 ng/ml) and bFGF (10 ng/ml) (R&D Systems). GICs were grown to subconfluence, dissociated using TrypLExpress (Invitrogen), and then split 1∶2–1∶4. 2/3 of medium was replaced with fresh medium every 3–4 days. For differentiation, cells were cultured in DMEM/F12 media supplemented with 10% fetal bovine serum (FBS; Gibco, Life Technologies)) for two weeks

Novel human malignant glioblastoma initiating cell (GIC) cultures (U3NNN-MG series of GIC lines) used in this study are part of the Uppsala University Human Glioma Cell Culture (HGCC) collection, which comprises well-characterized GBM-derived cancer initiating cell cultures (Xie et al 2014, manuscript in preparation). This work was approved by the Uppsala ethical review board (2007/353). All GIC lines were used between passages 15 and 30.

### Cell assays

GliNS1, G179NS and G166NS GIC lines, both undifferentiated and differentiated, were seeded on day 1 at 20% density onto laminin-coated 96 or 384 black well, flat bottom microplates (Corning). Compounds were added to the plates on day 2, followed by incubation for 48 hrs. FBS differentiated cells received serum-free media (50% human Neurocult NS-A basal media, 50% Neurobasal media (Gibco, Life Technologies), supplemented with Glutamax, Hepes, B27, ¼ N2, no growth factors) during chemical compound treatment. DMSO was used as negative control. Viability assay was performed using the CellTiterGlo assay (Promega, Madison, WI) according to the manufacturer's recommendations. Briefly, assay reaction buffer was added to the wells using an automated multipipette, followed by shaking the microplate for 30 seconds and 7 min incubation in the dark. Luciferase intensity reading was then taken using Victor2 (Perkin Elmer) with a setting of 1 s luminescence reading per well. Z-factor was calculated for each experiment. For every cell line (GliNS1, G179NS, G166NS and hf5205NS), at least 3 replicates were analyzed. Statistical calculations were performed with GraphPadPrism (GraphPad Software, Inc. San Diego, CA, USA). Dose-response data were processed using log-linear interpolation to obtain log IC_50_ values.

Drug assays in novel GIC lines (U3NNN-MG series) were seeded in 384-well microplates (BD Falcon Optilux Cat. #353962) 24 hours prior to treatment using a Multidrop 384 liquid dispenser (ThermoScientific, Sweden). To ensure growth phase at end of the assay (∼70% confluency) cells were seeded at a density ranging between 2000–4000 cells/well. Drugs were transferred using the ECHO550 non-contact liquid dispenser (Labcyte, USA) to a 384 V- bottom polypropylene plate. The drugs were then diluted in medium and transferred using the MDT 384 head on a Janus automated workstation (PerkinElmer, USA) to the cell plates. Drugs were tested in 11-point dose dilution series and assayed for viability after 72 hours of treatment on an EnVision Multilabel reader (PerkinElmer, USA) using resazurin (R7017, SigmaAldrich, Stockholm, Sweden), at the excitation/emission wave- length 560/590 nm [20]. As a positive control, the drug doxorubicin was screened with the same dose-response curve setting, and wells containing negative DMSO controls at 4 different concentrations were assayed as well. The effect on viability of each drug dose was calculated as a viability ratio W =  Ytreated/Ycontrol, where Y represents the average fluorescence signal.

### RNA extraction, transcriptome and data analysis

Two replicates were analyzed for the undifferentiated and differentiated cell lines GliNS1, G166NS and G179NS, while three replicates were analyzed for DMSO, A23187 and Thapsigargin treated GliNS1 and G166NS. Total RNA was extracted from cells grown to subconfluency using the RNeasy Mini Kit (Qiagen), following the manufacturer's instructions. Fluorometric quantitation of RNA concentration and quality was done using the Qubit RNA assay kit (Invitrogen). We used 300 ng of total RNA in the preparation of the TruSeq library, for which we used the Illumina Low-Throughput TruSeq RNA Sample Preparation Kit protocol resulting in barcoded cDNA. 50 ng of barcoded TruSeq products were used for Illumina RNA sequencing. Samples were sequenced on an Illumina HiSeq 2000 sequencer as single-end 51-nucleotide reads according to the manufactures protocol. Raw reads were mapped to the reference human genome and normalized data was generated for each genomic feature using STRT software [21]. Briefly, raw reads were aligned using Bowtie [22]. Mapped reads were normalized using reads per KB per million reads (RPKM) normalization method [23] whereas unmapped reads were removed. Differential gene expression analysis was done in R-studio using the DESeq package [24] and a script adopted from a previous paper [25]. Benjamini adjusted p-values were used for data analysis. Data analysis was done using Qlucore Omics Explorer 2.0 (Qlucore AB), PRISM 6 (GraphPad Software), DAVID (http://david.abcc.ncifcrf.gov) and GeneVenn (http://genevenn.sourceforge.net).

The Uppsala U3NNN-MG cell lines were not analyzed in replicates. For each cell line total RNA was extracted from cultured cells using the RNeasy Mini kit (Qiagen) and was labeled and hybridized on Affymetrix GeneChip Human Exon 1.0 ST arrays following the instructions of the manufacturer. The expression values were RMAnormalized using the Affymetrix Expression Console software.

### Immunofluorescent staining

Cells cultured attached on cover glass were fixed in 4% paraformaldehyde (PFA) for 15 min at room temperature (RT) followed by antibody incubation at 4°C overnight. The following primary antibodies were used: rabbit anti-glial fibrillary acidic protein (GFAP) (DAKO Cytomation) and mouse anti-beta III tubulin (Tuj1) (Millipore). The cells were then incubated with fluorescence-labeled secondary antibodies anti-rabbit Alexa Fluor 555 and Alexa Fluor 488 (Invitrogen) for 1 hr at RT. Nuclei were counterstained with DAPI and mounted using Immumount (DAKO). Images of stained cells were acquired with an Olympus FV1000 confocal microscope and processed with Adobe Photoshop CS5.1 software.

### Western blot analysis

Cells were lysed in RIPA buffer and protein concentration was quantified using the Bicinchoninic Acid (BCA) Protein Assay Kit (SigmaAldrich; Pierce, Rockford, US). Equal amounts of protein samples were separated by electrophoresis using 10% Mini-PROTEAN TGX gels (Bio-Rad) or 4–12% Bis-Tris polyacrylamide gradient gel (NuPAGE, Invitrogen) under reducing conditions and transferred onto a PVDF membrane (Bio-Rad) or nitrocellulose membrane (Hybond-ECL, GE Healthcare, Sweden). Membranes were blocked in 5% milk for 1 hour and incubated overnight at 4°C with the following primary antibodies: rabbit anti-GRIA1 antibody (Abcam), rabbit anti-S100 alpha 6 antibody (Abcam), mouse anti-BLBP antibody (Millipore) and mouse anti-beta actin antibody (Abcam). The membranes were then incubated with the appropriate horseradish peroxidase-labeled secondary antibody (SigmaAldrich; GE Healthcare) before being revealed by chemiluminescence (Clarity Western ECL Substrate, Bio-Rad; SuperSignal West Pico Chemiluminescent Substrate (Pierce, Rockford, US)). The band intensities were quantified using ImageJ (NIH, USA).

## Results

### Transcriptome profiling identifies stemness-related Ca^2+^ gene expression in GICs

**Figure 1:**
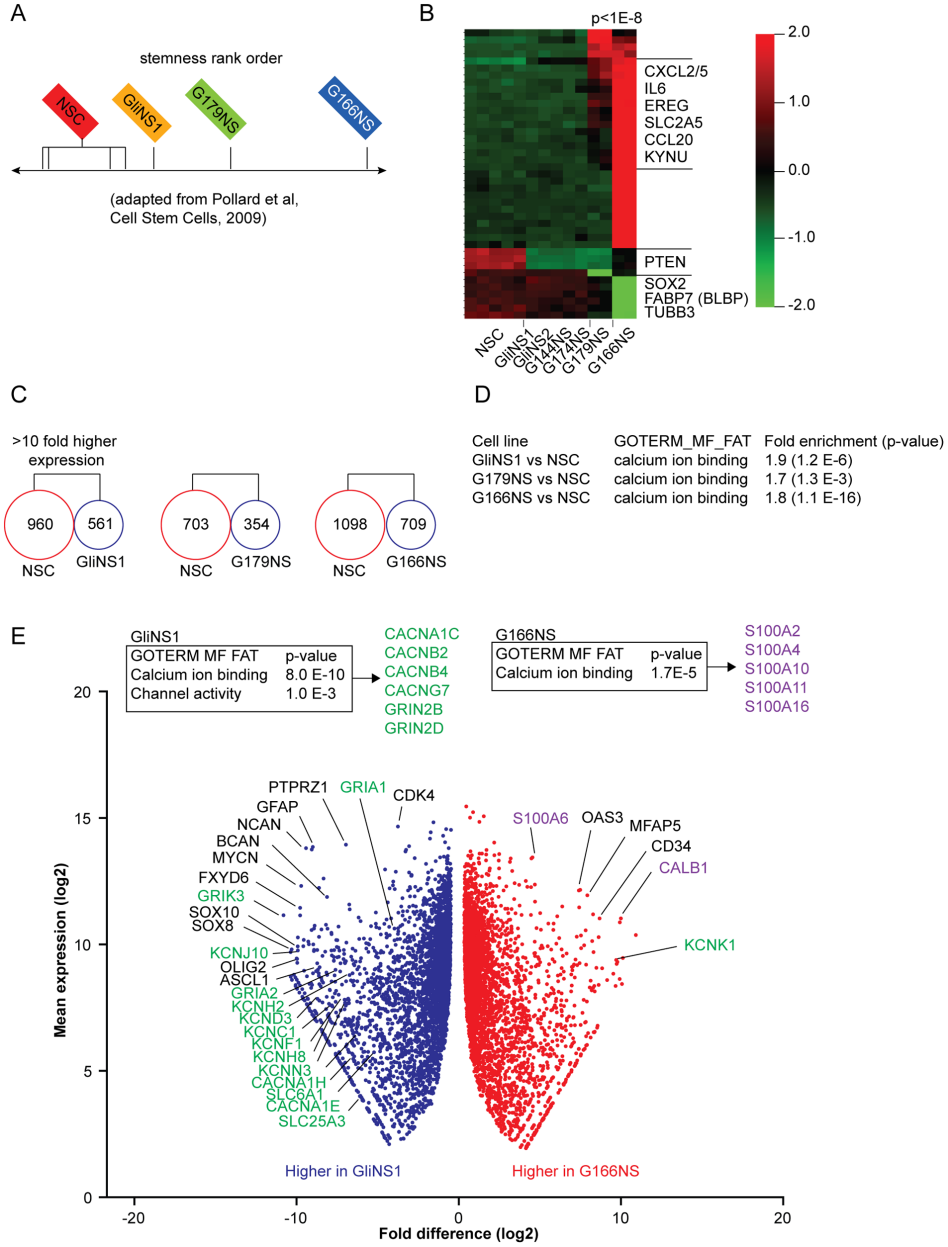
Expression of genes involved in Ca^2+^ signaling in GICs correlating with a NSC-associated transcriptome. (A) GIC lines rank ordered in relation to NSC lines (second component in a principle component analysis of microarray based mRNA expression data from Pollard et al [11], where the first component segregates NSCs and GICs from normal brain tissue). GliNS1 is derived from the G144ED line in the Pollard et al study. (B) Re-analysis of transcriptome profiles in Pollard et al comparing GICs to NSCs indicating a NSC-proximal cluster of stem-like GICs with high similarity to NSCs, sharing e.g. SOX2 and BLBP expression. NSC-distal GIC lines in contrast expressed microglia markers, such as CXCL2, CXCL5 and CCL20. (C) De novo RNA sequencing analysis and pairwise comparisons of NSCs and three individual GIC lines (GliNS1, G179NS and G166NS) showed that NSCs expressed a larger number of genes with 10-fold higher gene expression compared to all GIC lines. (D) Pairwise comparisons of NSCs to the GIC lines GliNS1, G179NS and G166NS, individually. Gene enrichment and gene ontology analysis of sequencing based transcriptome profiles, identified an enrichment of Ca^2+^ signaling genes in NSCs, which increased with rank order distal to NSC in pairwise comparisons. (E) Pairwise comparisons of the NSC-proximal (GliNS1) and NSC-distal (G166NS) GICs. Gene enrichment and gene ontology analysis suggested a switch in Ca^2+^ permeable channels to Ca^2+^ binding genes in the NSC-distal GIC line (upper boxes). In volcano plot, gene names in green denote ion channel/pump/transporter related genes, whereas gene names in purple denote Ca^2+^ binding proteins genes. The volcano plot of the comparison of NSC-proximal and NSC-distal GICs revealed a larger number of ion channels expressed in the NSC-proximal GIC (GliNS1).

To determine the relationship between human GIC lines and human fetal neural stem cells (NSC), we re-analyzed the microarray data from a previous study by Pollard *et al* [11]. While the first principal component segregated normal brain from NSCs and GICs (see [11]), the second principal component ranked GICs in relation to their similarity to NSCs, potentially reflecting aspects of stemness (Fig. 1A). The stemness-associated gene SOX2, the NSC marker BLBP (brain lipid-binding protein, encoded by the FABP7 gene) as well as the neuronal marker TUBB3 (Tubulin beta III), which may reflect high potency for concomitant neuronal differentiation, were expressed the highest in NSCs, and expression levels decreased in the order of the GliNS1 group > G179NS > G166NS (Fig. 1B). While the GliNS1 line was transcriptionally related to NSCs (NSC-proximal), the G166NS line, constituted the distal end of the ranking relative to NSCs (NSC-distal), expressing markers shared with microglia and reactive astrocytes and associated with inflammation, for example IL-6, CXCL2 (MIP-2), and CCL20 (Fig. 1B).

A de novo deep RNA sequencing of three of the GIC lines used in the Pollard *et al* study (GliNS1, G179NS and G166NS) and a NSC line (hf5205NS) showed that more genes were expressed in NSCs than GIC lines (Fig. 1C; S1), potentially reflecting their plasticity and ability to differentiate. Pairwise NSC-GIC gene enrichment and functional annotation (gene ontology) analysis unexpectedly showed that Ca^2+^ ion binding was the most significantly altered category in all three cell lines - a difference that increased in more NSC-distal GIC lines (Fig. 1D).

### Differential expression of Ca^2+^ provokers and buffers relates to stemness

Cytosolic Ca^2+^ signaling is balanced by various players, such as Ca^2+^ permeable ion channels that increase intracellular Ca^2+^ (e.g. voltage gated Ca^2+^ channels and glutamate receptors, here termed Ca^2+^ provokers) on one hand, and Ca^2+^ binders that reduce free intracellular Ca^2+^ on the other (here termed Ca^2+^ buffers) [26]. Direct pairwise comparisons between different GIC lines showed that the NSC-proximal GIC line (GliNS1) expressed a larger number of ion channel genes that include Ca^2+^ provokers compared to the NSC-distal GIC line (G166NS). These ranged from Ca^2+^ permeable ion channels (e.g. glutamate receptors: GRIA1, GRIA2, GRIK3 and regulatory subunits GRIN2B and GRIN2D), voltage-gated Ca^2+^ ion channels (CACNA1C/-E/-H) and Ca^2+^-activated potassium channels (e.g. KCNN3) (Fig. 1E). In contrast, the NSC-distal GIC line (G166NS) expressed higher levels of sensors of intracellular Ca^2+^ buffers (for example S100 family genes and CALB1).

**Figure 2:**
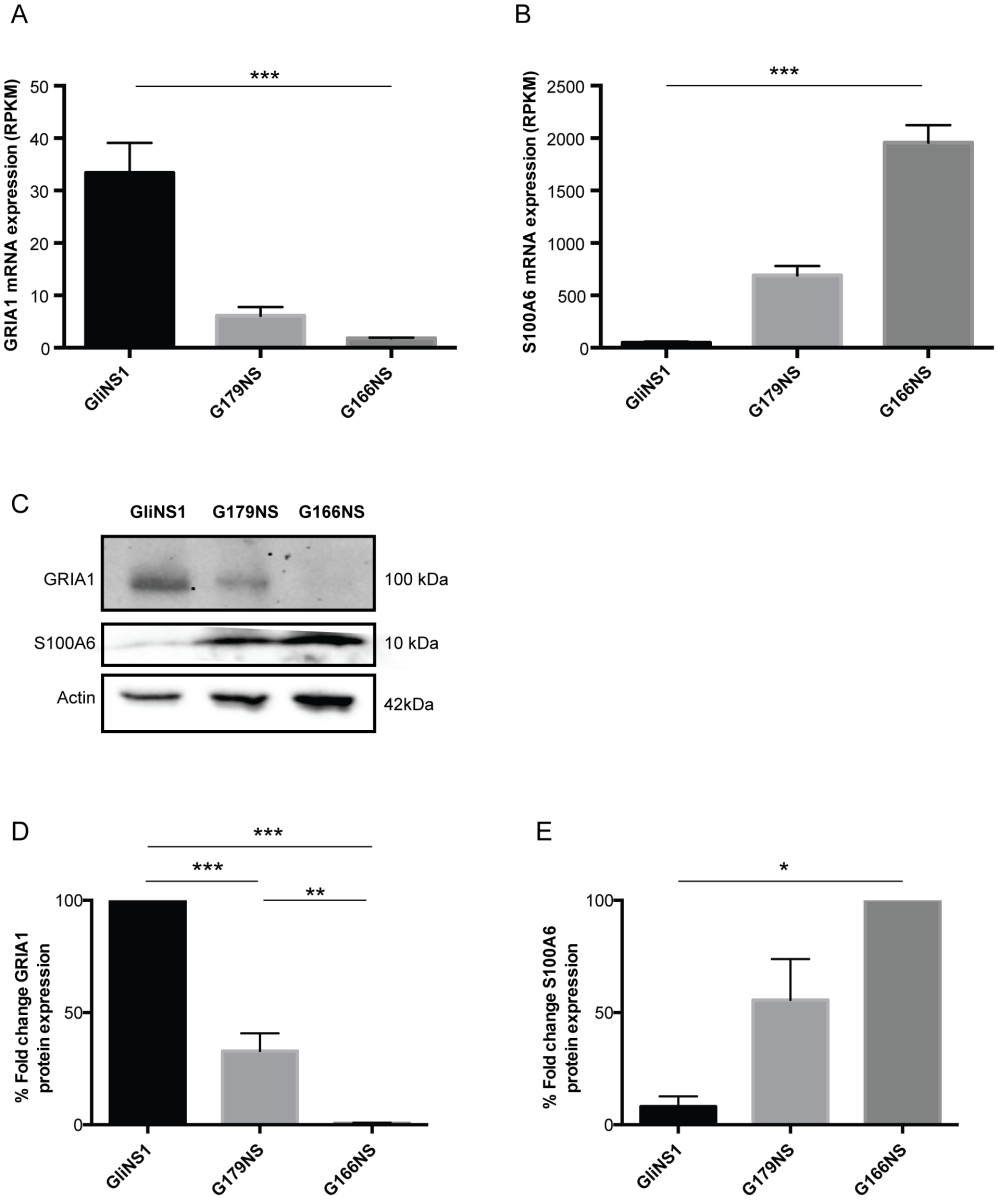
Ca^2+^ provoker and buffer expression in NSC-proximal and NSC-distal GIC lines. Analysis of expression of Ca^2+^ provokers such as one of the permeable glutamate receptor subunits GRIA1 (A) or Ca^2+^ buffer S100A6 (B), ranked the 3 GIC lines (GliNS1 and G166NS, n = 5 each. G179NS, n = 2.) according to Ca^2+^ drug sensitivity, with glutamate channels, such as GRIA1, predicting higher sensitivity and buffer expression predicting lower sensitivity (***p<0.001; Unpaired two-tailed t-test). (C) Western blot analysis showed GRIA1 and S100A6 protein expression, with β-actin as loading control. (D) Protein expression levels of GRIA1 were expressed as percentage fold change when compared against to GliNS1 (n = 3) and (E) S100A6 protein expression levels were expressed as percentage fold change when compared against G166NS (n = 3) (***p<0.001; **p<0.01, *p<0.05 One-way ANOVA).

To further delineate differences in Ca^2+^ gene expression between tested GIC lines and NSCs, expression of Ca^2+^ provokers and buffers was analyzed in more detail in all three GIC lines (S1 Fig.). As suggested in the previous pairwise comparisons, expression of glutamate receptors decreased in NSC-distal GIC lines (Fig. 2A). The AMPA receptor GRIA1 (Ca^2+^ permeable ionotropic glutamate receptor), which showed the highest expression among glutamate receptors, ranked the GIC lines identically to the order GliNS1> G179NS> G166NS seen in the PCA analysis (Fig. 1A) based on comparison with NSC gene expression. In contrast, expression of Ca^2+^ buffers/effectors increased with an inverse rank order of GliNS1 <G179NS <G166NS (Fig. 2B). This was particularly striking for the S100A6 gene that was most abundantly expressed in G166NS. Similar to the mRNA data, western blot analysis of GRIA1 revealed a 70% and more than 95% lower expression in G179NS and G166NS respectively when compared to the NSC-proximal GliNS1 line (Fig. 2B and 2E). The western blot analysis of S100A6 showed a 45% and 90% lower expression in G179NS and GliNS1, respectively, when compared to the NSC-distal G166NS line (Fig. 2C, 2D and 2E).

### GIC Ca^2+^ drug sensitivity correlates with transcriptome proximity to NSCs

**Figure 3:**
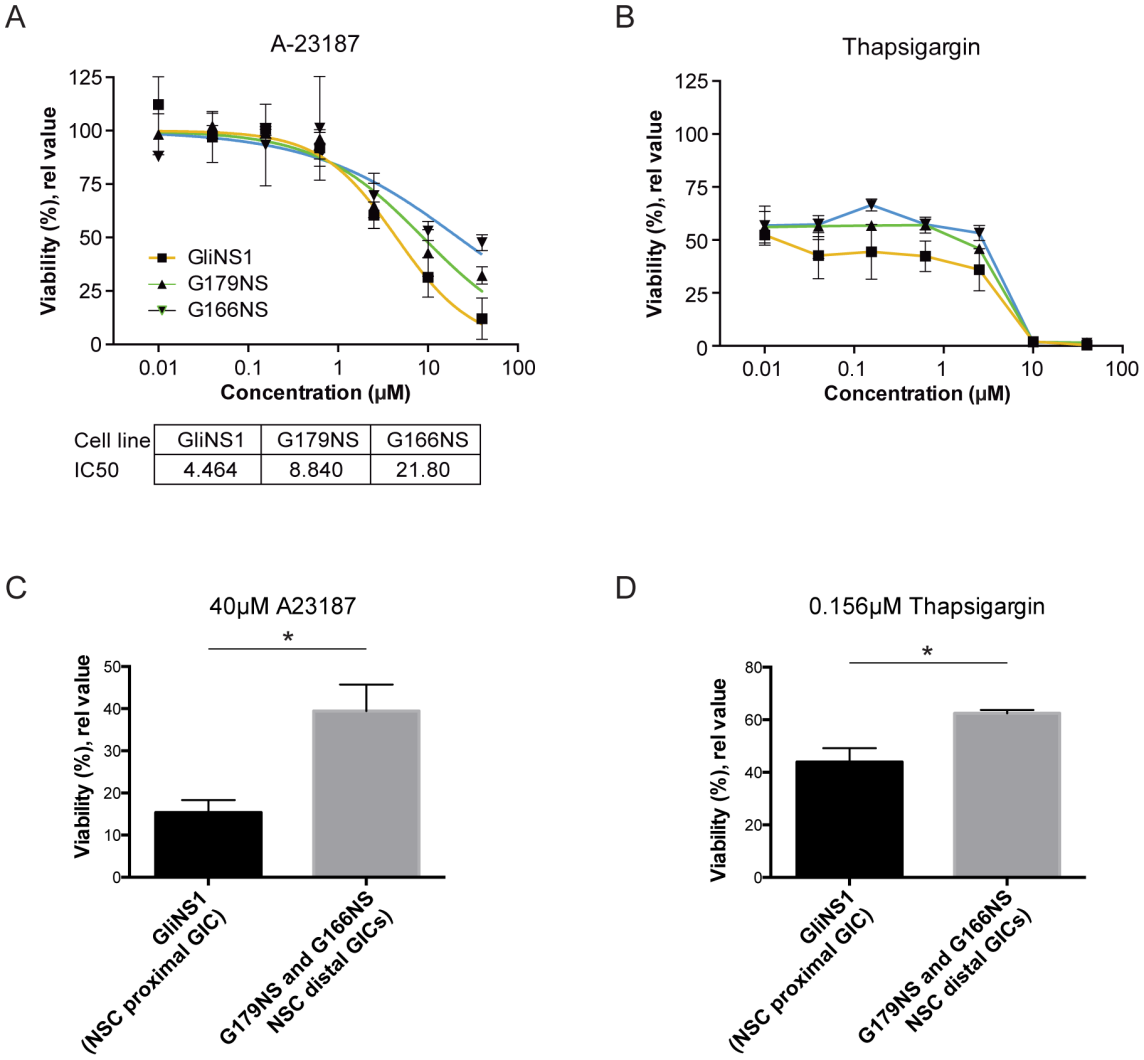
Sensitivity to drugs targeting Ca^2+^ homeostasis follows GIC transcriptome rank order relative to NSCs. (A) Dose response analysis (0.01–40 µM) of the Ca^2+^ ionophore A23187 and (B) the SERCA Ca^2+^ pump inhibitor Thapsigargin showed that Ca^2+^ drug sensitivity rank ordered with transcriptome similarity to NSCs, with highest sensitivity in the NSC-proximal GICs. NSC proximal GIC was more sensitive to (C) 40 µM A23187 and (D) 0.156 µM Thapsigargin treatments as compared to the NSC distal lines (*p<0.05; Unpaired two-tailed t-test). NSC-proximal GICs n = 3 and NSC-distal GICs n = 4.

In addition Ca^2+^ provokers and buffers, plasma membrane localized Ca^2+^ transporters, such as sodium-calcium exchangers (NCX) belonging to the SLC8 family and the SERCA (sarco/endoplasmic reticulum Ca^2+^ ATPase) pump localized in the endoplasmic reticulum (ER) membrane, actively remove cytosolic Ca^2+^ to maintain homeostasis [27]–[30]. To explore potential functional implications of a differential expression of Ca^2+^ ion channels and Ca^2+^ binding genes, we next performed a drug-mediated challenge of Ca^2+^ homeostasis and signaling, in the GIC lines. Cells were exposed to either to the target independent cation (Ca^2+^) ionophore A23187 or the SERCA pump inhibitor Thapsigargin (Fig. 3), which increase cytosolic Ca^2+^ levels by two different mechanisms: A23187 by allowing Ca^2+^ to cross the normally impermeable cell membrane, and Thapsigargin by blocking import of Ca^2+^ into the ER. The GIC lines showed differences in sensitivity for both A23187 and Thapsigargin (Fig. 3A and 3B, respectively), remarkably with a rank order between the lines identical to that of the NSC-rooted transcriptome rank order, with the NSC-proximal GliNS1 being more sensitive than G179NS, while the NSC-distal G166NS was least sensitive to both drugs. Functional analyses thus show that NSC-proximal GICs with a higher expression of Ca^2+^ provokers, are more sensitive to disturbances in cytosolic Ca^2+^ regulation than GICs with a NSC-distal phenotype that express higher levels of Ca^2+^ buffers.

### Reduced Ca^2+^ drug sensitivity upon GIC differentiation

**Figure 4:**
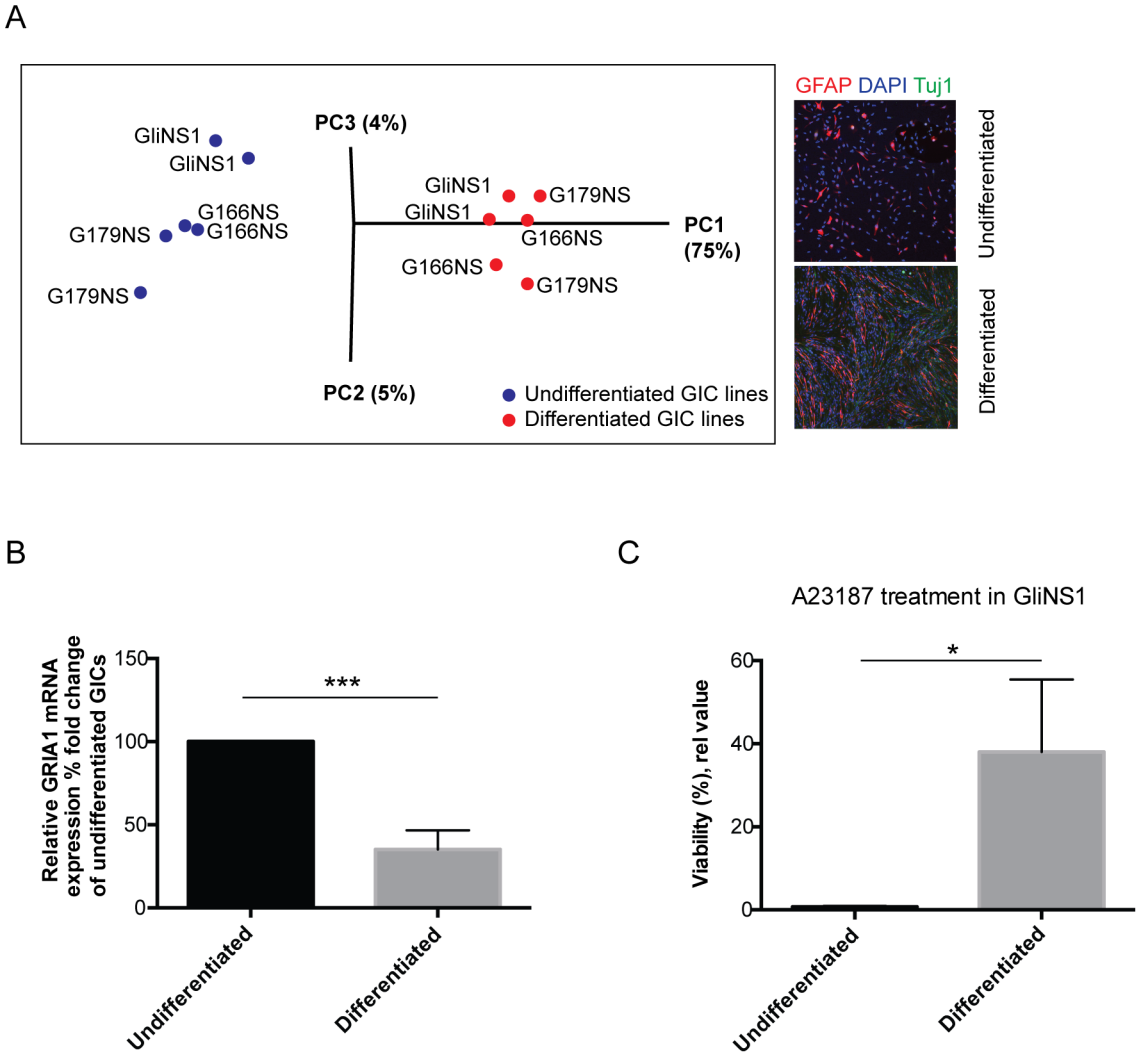
Decreased sensitivity to A23187 during GIC differentiation correlating with decrease in GRIA1 expression. (A) RNA sequencing transcriptome mapping followed by principal component analysis verified segregation between undifferentiated and differentiated GICs (GliNS1, G179NS and G166NS). Right panel shows immunofluorescent stainings of the differentiation markers GFAP (red) and Tuj1 (green) upon FBS treatment. (B) Comparison of GRIA1 expression levels in undifferentiated and differentiated GICs (n = 6) revealed a reduction in fold change in GRIA1 expression upon serum-induced differentiation in all GIC lines. (***p<0.001; Unpaired two-tailed t-test). (C) Cell viability analysis of relative sensitivity to the Ca^2+^ ionophore A23187 after differentiation showed increased viability upon differentiation of the NSC-proximal GIC line GliNS1 (*p<0.05; Unpaired two-tailed t-test).

As sensitivity to Ca^2+^ drugs was associated with a NSC-like expression profile the question whether differentiation of GICs would affect Ca^2+^ sensitivity was investigated. To this end, three GIC lines were subjected to a differentiation protocol using fetal bovine serum (FBS). Validation of differentiation was done by transcriptome analysis of the GIC lines and their differentiated progeny using RNA sequencing (S1 Table). Principal component analysis of the global data set showed that changes in the transcriptome were distinct and segregated significantly between undifferentiated GICs and differentiated GICs (diffGICs) (Fig. 4A). Interestingly, GRIA1 expression that correlated with Ca^2+^ drug sensitivity, decreased in all GIC lines during differentiation (Fig. 4B), which suggested that differentiation status might affect Ca^2+^ sensitivity. Functional Ca^2+^ sensitivity was therefore assayed using A23187 (10 uM, 48 hours) in differentiated GICs and compared to undifferentiated GICs revealing a clearly reduced effect on cell viability in all GIC lines upon differentiation and with the strongest effect in the drug sensitive NSC-proximal GIC line (GliNS1)(Fig. 4C). These findings further support the data that Ca^2+^ sensitivity is associated with immature NSC-like GICs.

### Gene expression correlating with Ca^2+^ drug sensitivity

**Figure 5:**
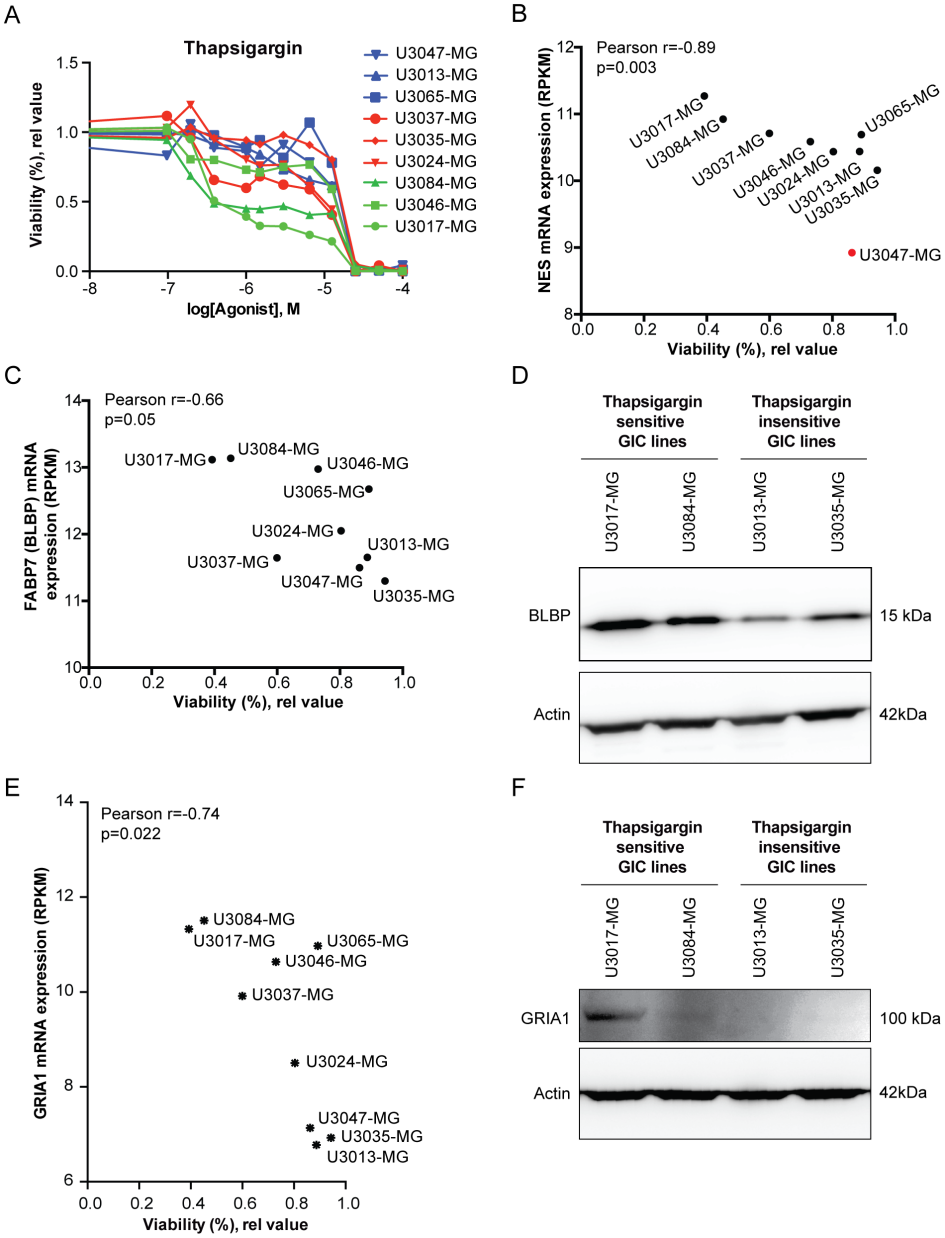
Genome wide correlation analysis between Ca^2+^ drug sensitivity and gene expression. (A) Nine novel GIC lines were subjected to Thapsigargin dose response analysis (0.1–100 µM), showing different response to moderate drug doses. (B, C) Plot of correlation between cell viability after Ca^2+^ drug exposure (Thapsigargin, 1 uM) and (B) NES and (C) FABP7/BLBP mRNA expression. U3047-MG was considered an outlier in the NES graph (marked in red) and excluded form the analysis. (D) Western blot analysis showing BLBP (FABP7) protein expression in selected Thapsigargin sensitive (U3017-MG and U3084-MG) and less sensitive (U3013-MG and U3035-MG) cell lines, with β-actin as loading control. (E) Plot of correlation between cell viability after Ca^2+^ drug exposure (Thapsigargin, 1 uM) and GRIA1 mRNA expression. (F) Western blot analysis showing GRIA1 protein expression in selected Thapsigargin sensitive (U3017-MG and U3084-MG) and less sensitive (U3013-MG and U3035-MG) cell lines. β-actin was used as loading control.

**Figure 6:**
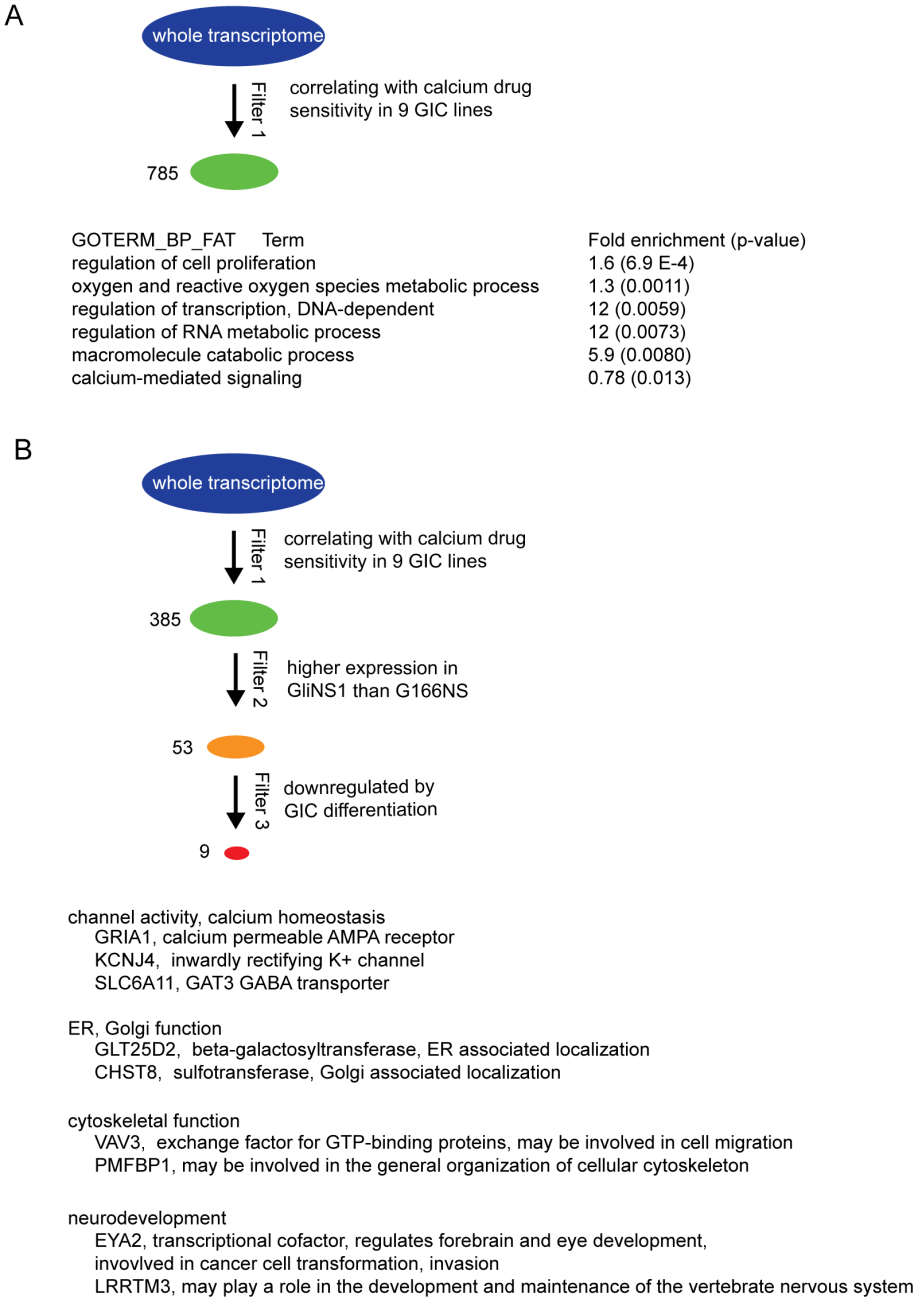
Gene expression correlating with high Ca^2+^ sensitivity in 9 GIC lines. (A) A correlation analysis of genome wide mRNA expression (microarray analysis) and sensitivity to Thapsigargin (1 uM) in 9 additional GIC lines, retrieved 785 genes correlating with Ca^2+^ drug sensitivity. Gene enrichment and ontology analyses identified involvement of genes affecting proliferation, oxygen and RNA metabolism, catabolism and Ca^2+^-mediated signaling. (B) 385 genes positively correlating with high sensitivity were filtered first for genes also expressed higher in the NSC-proximal GIC line GliNS1 and thereafter also being downregulated in this line upon differentiation, which was found to reduce Ca^2+^ drug sensitivity, retrieving a set of nine genes, including the AMPA receptor coding GRIA1.

To explore potential additional genes correlating with Ca^2+^ sensitivity, transcriptome data from nine novel GIC lines (derived in an independent laboratory) was compared to Ca^2+^ sensitivity data from exposure to Thapsigargin (72 h exposure)(Fig. 5A and S2 Table). 7 out of the 9 lines have been shown to recapitulate the parent tumor (S3 Table). Analysis of correlation (Pearson) between NSC-markers and sensitivity to Thapsigargin (1 uM) revealed a significant correlation for nestin (NES) (Fig. 5B, outlier marked in red was excluded from the analysis) and brain lipid-bindig protein (BLBP/FABP7) (Fig. 5C) mRNA expression, while no correlation was found for SOX2 (data not shown). Western blot analysis further verified that calcium drug sensitive lines (U3017-MG and U3084-MG) expressed more BLBP protein than less sensitive lines (U3013-MG and U3035-MG) (Fig. 5D). The correlation analysis also confirmed a correlation between sensitivity to Thapsigargin and GRIA1 expression (Fig. 5E), which was corroborated by analysis of protein levels by western blot, as GRIA1 protein expression was only detected in the sensitive GICs (Fig. 5F). Further gene enrichment and gene ontology analyses implied genes involved in cell cycle regulation, oxygen, RNA and macromolecule metabolism, and not unexpectedly Ca^2+^-mediated signaling as correlating with Ca^2+^ drug sensitivity (Fig. 6A).

To identify genes in this data set that also associated with a NSC-proximal stemness signature in GICs, the set was further filtered for genes, which also (1) had a higher expression in GliNS1 compared to G166NS and (2) were downregulated upon differentiation (Fig. 6B). This retrieved a short-list of nine genes, two of which code for ion channels that may increase cytosolic Ca^2+^ (Ca^2+^ provokers), i.e. GRIA1 and the inward rectifier K^+^ channel KCNJ4, which may participate in maintaining a depolarized membrane potential required to activate voltage-gated Ca^2+^ channels and Ca^2+^ permeable glutamate receptors. In summary, the correlation between functional Ca^2+^ drug sensitivity and gene expression suggests participation towards sensitivity to drug-elicited Ca^2+^ overload, by a network of genes involved in maintaining Ca^2+^ homeostasis and membrane potential.

### Drug reactome analysis identifies Ca^2+^-induced gene expression in the global transcriptome

**Figure 7:**
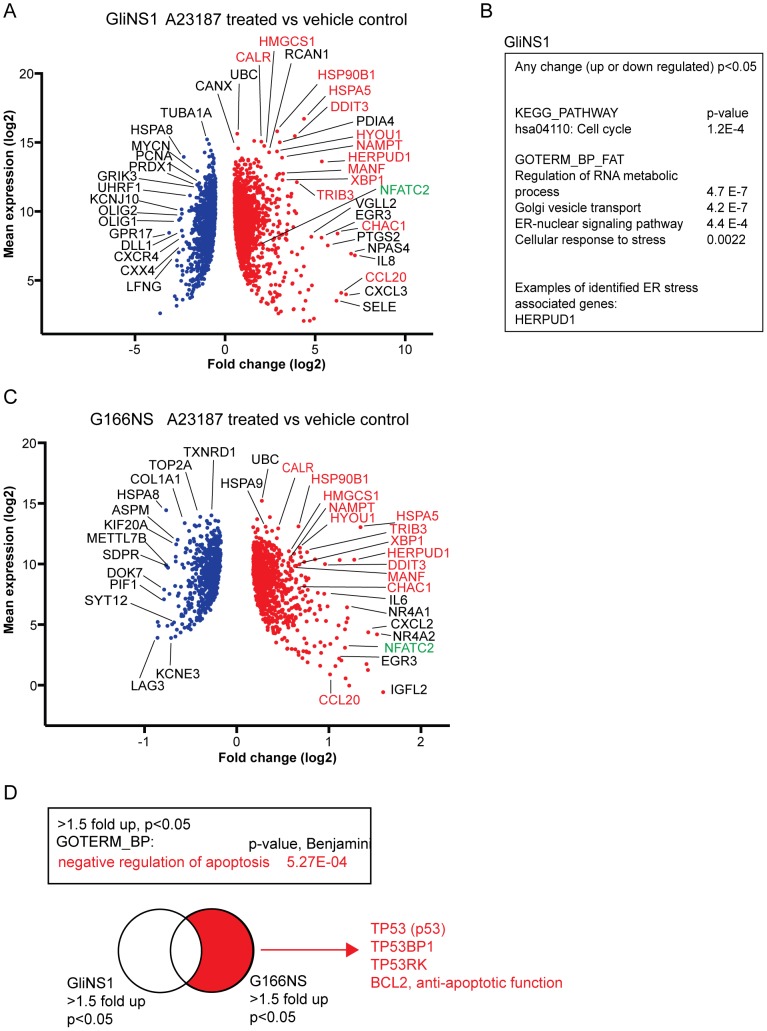
Transcriptome analysis of drug response in GliNS1 and G166NS. Transcriptional response to increased cytosolic Ca^2+^ (A23187), was investigated by RNA sequencing after 7 hours of drug exposure in the NSC-proximal GIC line GliiNS1 and the NSC-distal line G166NS. Volcano plots of significantly (p<0.05) altered gene expression in GliNS1 (A) and G166NS (C) with shared induced genes marked in red and green (Ca^2+^ activated transcription factor NFATC2). Note the differences in x-axis indicating higher all global induction of gene expression in GliNS1. (B) Gene enrichment and gene ontology analysis of genes with a significant change in expression (p<0.05) in GliNS1, identified genes involved in cell cycle progression as well as ER/golgi associated functions and cellular stress response. (D) Gene enrichment analysis of genes downregulated at least 3-fold in GliNS1 and upregulated at least 1.5-fold in G166NS.

To identify intracellular responses to Ca^2+^ underlying the differential level of Ca^2+^ sensitivity in GICs, the NSC-proximal GliNS1 and NSC-distal G166NS were exposed to A23187 for 7 hours, followed by transcriptome analysis by RNA sequencing (“drug reactome mapping”)(S4 Table). In the most Ca^2+^ drug sensitive GIC line GliNS1, genes with significantly altered expression (Benjamini adjusted p-value <0.05) were analyzed by gene enrichment and gene ontology, which showed that cell cycle related genes were altered (Fig. 7B and S2 Fig.), suggesting cell cycle arrest prior to cell death. Not unexpectedly, genes involved in ER stress response were also enriched, as were genes in RNA metabolic processes. Interestingly, RNA metabolic process involved genes were also correlating with Thapsigargin sensitivity in the previous experiment (Fig. 6A).

Genes with altered expression after drug exposure were plotted against mean expression value (before drug exposure) to identify robustly altered genes with a potential biological significance. Strikingly, the GliNS1 line induced a clearly higher global transcriptome fold change than the less sensitive G166NS (Fig. 7A and 7C) suggesting a more potent onset of Ca^2+^ signaling in sensitive GICs. This may be the consequence by an inability to effectively reduce cytosolic Ca^2+^ levels. Interestingly, a very similar set of genes were altered in both the NSC-proximal (GliNS1) and the NSC-distal (G166NS) GIC lines, including Ca^2+^-binding genes acting as buffers (e.g. HSPA5 and HSP90B1) and Ca^2+^ related ER stress response (e.g. HERPUD1 and CALR). Also Ca^2+^-activated transcription factors (e.g NFATC2) were induced in both lines (Fig. 7A and 7C), suggesting that increased cytosolic Ca^2+^ could trigger a positive feedback mechanism. Interestingly, the G166NS line uniquely upregulated a set of anti-apoptotic genes, such as TP53 (coding for p53) and BCL2 genes (Fig. 7D), providing an explanation for lower sensitivity to e.g. Thapsigargin in addition to lower expression of Ca^2+^ permeable ion channels and higher expression of Ca^2+^ buffers.

## Discussion

Our study identified a stemness-associated sensitivity in GICs related to Ca^2+^ homeostasis and signaling. This finding was functionally validated by increasing cytosolic Ca^2+^ using the ionophore A23187, which causes uncontrolled uptake of Ca^2+^ over the plasma membrane, and Thapsigargin, which causes elevated cytosolic Ca^2+^ via depletion of Ca^2+^ stored in ER. GICs with a NSC-like transcriptome profile were more sensitive to disrupted Ca^2+^ homeostasis and undifferentiated GICs were more sensitive than differentiated GICs. The degree of similarity in the transcriptome profile of individual GIC lines and NSCs correlated with Ca^2+^ sensitivity. In contrast, NSC-distal GICs, expressing markers of reactive astrocytes and microglia, expressed higher levels of Ca^2+^ buffers and were comparatively less sensitive to Ca^2+^ drugs. Ca^2+^ signaling is well known to be critical for normal somatic cells and embryonic [31] and adult stem cells [32] as well as cancer cells, controlling diverse functions, such as proliferation, migration and apoptosis [26], [33]. However, the elevated sensitivity to Ca^2+^ that we identified appears to correlate uniquely to stemness (e.g. expression of nestin and BLBP), suggesting that certain functions in NSC-like immature cancer cells, are affected differently by perturbing Ca^2+^ homeostasis than are more differentiated mature cells.

An important component in the observed Ca^2+^-sensitivity appeared to be capacity to reduce cytosolic Ca^2+^, either via lower expression of Ca^2+^ channels or via expression of Ca^2+^ buffers, such as S100 proteins. In this context the AMPA receptor subunit GRIA1 appeared as an interesting marker predicting sensitivity and potentially involved mechanistically in increasing sensitivity by allowing Ca^2+^ influx in response to glutamate. GRIA1 is a marker of GICs, as previously published [34], and we validate this finding by showing that GRIA1 is downregulated upon differentiation. This suggests that a network of genes involved in maintaining Ca^2+^ homeostasis and membrane potential in immature cells, may contribute to the observed differential sensitivity to Ca^2+^ overload.

Interestingly, Ca^2+^ drug reactome analysis identified upregulated expression of a set of Ca^2+^ binding proteins and Ca^2+^-activated transcription factors, suggesting a positive feedback loop of Ca^2+^-responsive transcription that may be expected to further increase a Ca^2+^ transcriptional response. This is somewhat surprising, unless Ca^2+^ transcriptional response also includes functions to reduce or abort an intracellular calcium surge. A comparison between GliNS1 and the slightly less Ca^2+^ sensitive GIC line G166NS was made to potentially identify genes that may correlate with Ca^2+^ overload and hence may protect the cells. The drug reactome profile unique for G166NS identified genes protecting from apoptosis, thus contributing to the relative lower sensitivity to Ca^2+^ overload and giving an alternative explanation than interplay between Ca^2+^ provokers and buffers.

We recently reported a novel target in GICs critical for cell volume homeostasis [14]. However reports of GIC selective drug target are scarce and large scale chemical screens of GICs did not identify compounds that potently and selectively affected viability in GICs [11]. Stemness-associated Ca^2+^ sensitivity may be a novel such target with ability to eradicate a potentially malignant subpopulation in brain tumors. In this context, nestin, BLBP and GRIA1 are potential biomarkers predicting sensitivity to this drug in brain tumor cells.

## Supporting Information

S1 Fig **Analysis of expression of Ca^2+^ provokers such as permeable glutamate receptor subunits (A) or Ca^2+^ buffers (B) in GliNS1, G179NS and G166NS.**
(TIF)

S2 Fig **Overview of genes involved in cell cycle progression with direction of altered expression indicated in red (upregulation) or green (downregulation).**
(TIF)

S1 Table **Illumina mRNA sequencing data in simple reads of undifferentiated and differentiated GICs.**
(XLSX)

S2 Table **Top hits of pearson correlation analysis of genes and sensitivity to Thapsigargin in novel GIC cell lines.**
(XLSX)

S3 Table **A list of the novel GIC cell lines, their tumor initiating property and the median survival in mice (n = 5–10 mice per line).**
(XLSX)

S4 Table **Differential gene expression analysis of vehicle control vs A23187 treated GliNS1 and vehicle control vs A23187 treated G166NS.**
(XLSX)
