# Supplementary figures and images for: Selective Calcium Sensitivity in Immature Glioma Cancer Stem Cells
Source: PLoS One. 2014 Dec 22;9(12):e115698. doi: 10.1371/journal.pone.0115698 (PMC4274094; doi:10.1371/journal.pone.0115698)

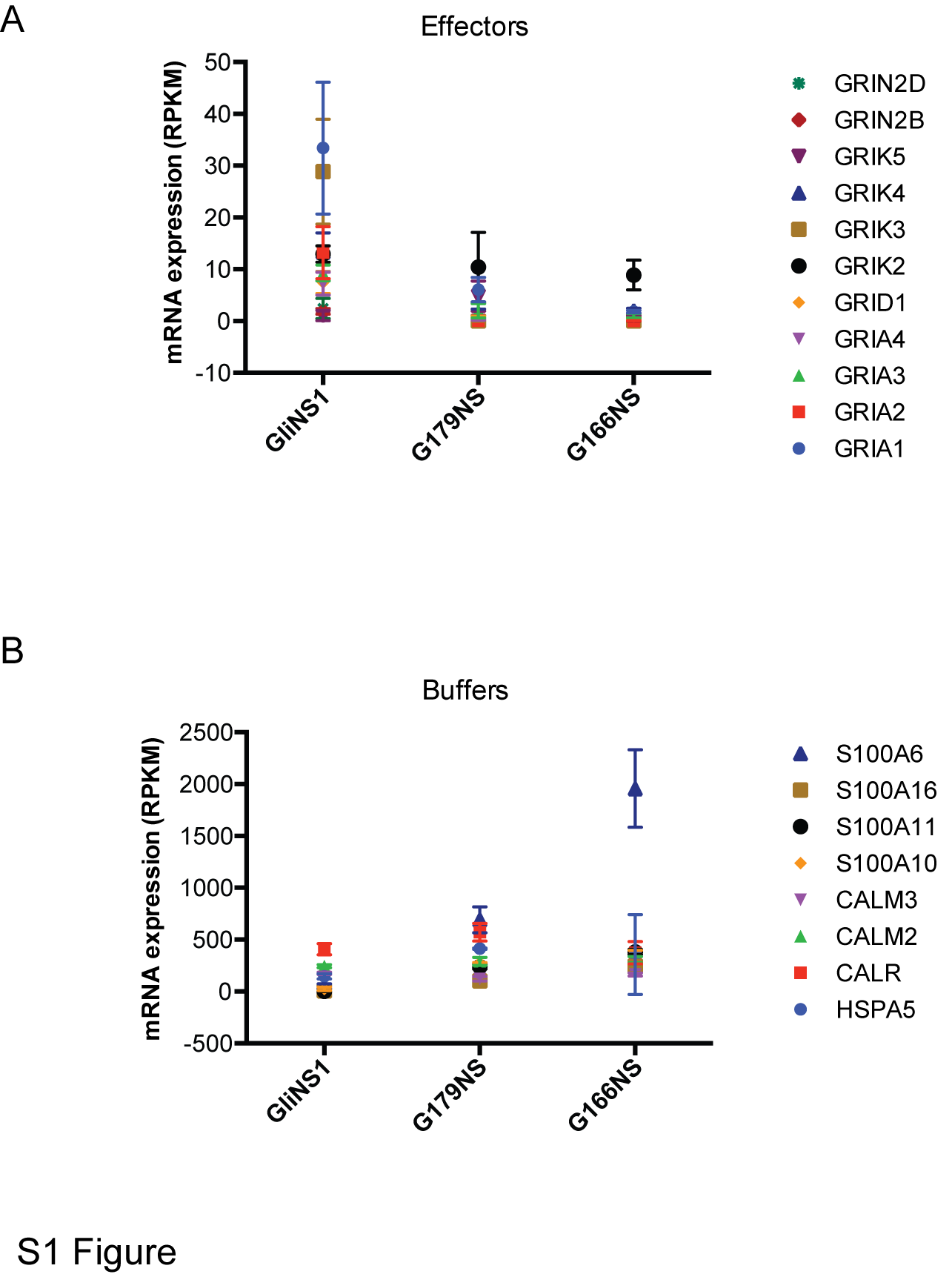

Supplement: S1 Fig — Analysis of expression of Ca2+ provokers such as permeable glutamate receptor subunits (A) or Ca2+ buffers (B) in GliNS1, G179NS and G166NS. (TIF) [file pone.0115698.s001.tif]

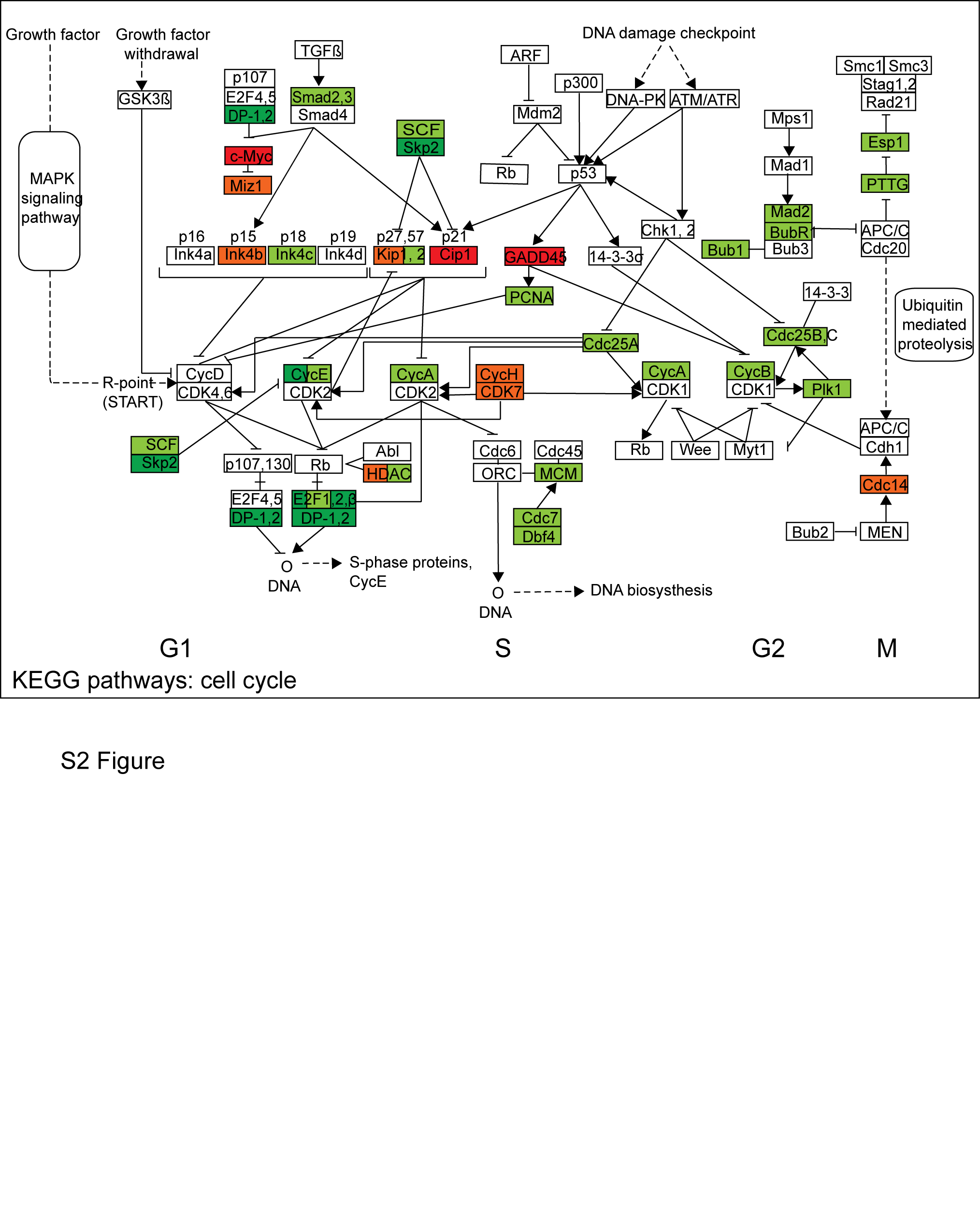

Supplement: S2 Fig — Overview of genes involved in cell cycle progression with direction of altered expression indicated in red (upregulation) or green (downregulation). (TIF) [file pone.0115698.s002.tif]
